# Person-centered care, shared decision-making, and service modularity in colorectal cancer treatment: A mixed-method study of patient and professional perspectives

**DOI:** 10.1371/journal.pone.0343331

**Published:** 2026-03-06

**Authors:** Mayke de Klerk, Evita A. Bartels, Janine Timmers, Lenny M. W. Nahar-van Venrooij, Angèle P. M. Kerckhoffs, Esther de Vries, Bert R. Meijboom

**Affiliations:** 1 Department of Tranzo Scientific Centre for Care and Wellbeing, Tilburg School of Social and Behavioral Sciences, Tilburg University, Tilburg, North Brabant province, The Netherlands; 2 Jeroen Bosch Academy Research, Jeroen Bosch Hospital, ‘s-Hertogenbosch, North Brabant province, The Netherlands; 3 Department of Information Systems and Operations Management, Tilburg School of Economics and Management, Tilburg University, Tilburg, North Brabant province, The Netherlands; 4 Department of Geriatrics, Jeroen Bosch Hospital, ‘s-Hertogenbosch, North Brabant province, The Netherlands; Mayo Clinic College of Medicine and Science, UNITED STATES OF AMERICA

## Abstract

**Background:**

Cancer care pathways improve outcomes through standardization but often lack flexibility to address individual needs. In this case study, we applied a composite model to examine its applicability by studying how person-centered care, shared decision-making, and service modularity are enacted within the colorectal cancer pathway of a large Dutch teaching hospital.

**Methods:**

We conducted a cross-sectional mixed-method single case study. Patients completed questionnaires on health-related well-being and colorectal cancer care experiences. We explored person-centered care, shared decision-making, and service modularity and their interaction using questionnaires (patients) and semi-structured interviews (patients and healthcare professionals). Quantitative data were analyzed with descriptive statistics; qualitative data were analyzed thematically.

**Results:**

Our findings showed gaps between formal structures and actual delivered care, especially in how shared decision-making and service modularity work together to provide person-centered care. Healthcare professionals’ limited modular thinking restricted customization. Information was delivered mainly according to protocols and did not fully address individual preferences, while preference elicitation was inconsistent. Care packages tended to follow clinical routines rather than reflect true patient priorities. Coordination was generally sufficient but revealed weaknesses during care transitions.

**Conclusion:**

The composite model provides a practical framework to enhance person-centered cancer care by revealing structural barriers to shared decision-making and customization. Promoting modular thinking among professionals supports flexible, preference-sensitive care and offers actionable strategies to improve both efficiency and patient engagement across oncological pathways.

## Introduction

The treatment of a patient with cancer is complex, requiring input from multiple healthcare professionals to address a patient’s cancer-related needs. Standardized, evidence-based treatment protocols aim to harmonize care quality of oncological clinical pathways. While these standardized approaches improve disease-related outcomes, they are not inherently tailored to individual patient needs, which extend beyond somatic aspects to social, psychological, and spiritual concerns, as well as management of co-existing non-malignant conditions. This underscores the need for a broader, more adaptive approach to cancer care.

In line with this, the concept of Positive Health, defined as the ability to adapt and self-manage in response to life’s challenges, has gained attention [1]. This concept shifts the focus from disease control to well-being across multiple domains: besides bodily functions, it includes mental health, a meaningful life, quality of life, participation, and daily functioning. Consequently, *person-centered care* considers the whole life context, supporting a meaningful life rather than only functional aspects [2].

Achieving truly person-centered care requires active patient involvement in care decisions. *Shared decision-making* supports this by promoting informed, value-aligned choices through structured dialogue between patients and healthcare professionals [3]. Yet, integrating shared decision-making and person-centered care into the rigid structure of oncological pathways remains challenging [4].

*Service modularity* is a promising approach to align standardization (ensuring uniform services for consistency, e.g., working evidence-based) with customization (tailoring services to individual needs and preferences) [5]. It decomposes healthcare services into interchangeable modules of components. Standardization at the component level enhances efficiency, while recombining modules allows tailoring to individual needs. Such a modular approach can enhance flexibility while preserving care quality without increasing costs. A modular perspective can also be used as an analytical lens to explore how healthcare services are organized, coordinated, and recombined across care pathways [6]. As Bartels et al. [7] argue, modularity’s full potential is only realized when it is embedded within a person-centered approach and applied in a shared decision-making context of the service specification process (the process in which the care package is composed). To translate this process into practice, Bartels et al. [7] proposed a modular care delivery model structured around five service specification phases: diagnosis and needs assessment, information exchange, care package composition, care provision, and follow-up. This model emphasizes structured patient involvement in each phase, allowing healthcare professionals to align standardized components with individual preferences and circumstances in collaboration with patients.

Colorectal cancer (CRC) care involves complex treatment decisions with profound implications for patients’ lives, making shared decision-making essential. The CRC pathway includes multiple treatment options, such as surgery, chemotherapy, radiotherapy, often in combination, depending on tumor size and tumor characteristics. Prehabilitation, aimed at improving patients’ physical and mental condition before treatment, is an integral part of this pathway to support better recovery and customized care [8]. While standardized protocols support clinical efficiency, they often lack the flexibility to address individual needs. Petersson et al. [9] highlighted tensions between standardization and customization in CRC pathways, underscoring the need for structured flexibility, interdisciplinary collaboration, and organizational support to ensure person-centered care. Modularity offers a way to support this by tailoring care packages to individual preferences and circumstances.

To examine how modularity can support customized oncology care in practice, we applied the Bartels et al. [7] composite model as a conceptual foundation to study current practice within the CRC pathway of the Jeroen Bosch Hospital (JBZ, a large teaching hospital in ‘s-Hertogenbosch, the Netherlands). This served two aims. First, we used the model in a real-world setting to explore its applicability as a form of theory-testing. Second, we examined how person-centered care, shared decision-making, and service modularity are perceived and enacted by both patients and healthcare professionals. By projecting the model onto current practice, we aimed to identify areas for improvement and to generate insights into how modular service design can better support meaningful, customized cancer care.

## Materials and methods

### Study design

We performed a cross-sectional mixed method single case study. Questionnaires were completed by patients to gain insight into the patients’ health-related well-being and experiences related to the CRC pathway. To further explore and contextualize these findings, semi-structured interviews identified experiences and issues related to the current CRC pathway. These interviews were conducted with CRC patients (patient perspective) and healthcare professionals (organizational perspective). The study design and data analysis were guided by the Consolidated Criteria for Reporting Qualitative Research (COREQ) [10].

### Setting

The study was performed at a large, teaching hospital (JBZ). The JBZ performs surgery on around 250 colon cancer patients per year and delivers advanced CRC care. Patients were recruited from 1 October 2019 to 1 May 2021. There were no significant changes in the healthcare system or staffing during the data collection period.

### Participants

The study population consisted of patients diagnosed with CRC and healthcare professionals involved in the CRC pathway. Patient recruitment occurred in two steps. For the questionnaires, patients were recruited convenience-based by a healthcare professional during consultations at the JBZ outpatient clinic. Eligible patients were at least 18 years of age, cognitively capable, able to provide informed consent, and attended at least three appointments at the JBZ related to their CRC. All patients initially underwent surgery followed by additional treatment as indicated within the CRC care pathway. The healthcare professional provided verbal information and a study leaflet to interested patients. Leaflets were also available in the outpatient waiting areas. Interested patients received an information letter and an informed consent form. Researcher EB remained available to provide further information in person or by telephone. Seventy-nine patients were approached to complete the questionnaires, of whom 65 patients gave their consent and were included (response rate 82%).

Patients who completed the questionnaire and indicated willingness to participate in an interview were subsequently contacted by researcher EB by telephone to request consent for an interview. Sixty-five patients were invited for an interview and 21 consented (38%). Three interviews were excluded from the analysis, two due to technical issues and one because the participant declined audio recording.

Healthcare professionals were recruited separately through purposive sampling; they were contacted by e-mail by researcher EB. All 24 invited professionals consented (response rate 100%). However, one interview was excluded due to the participant’s role falling outside the study scope as this participant was not part of the care network under study, resulting in 23 included professionals. There was no prior relationship between the researcher and the participants.

### Data collection

#### Quantitative data collection.

Four different questionnaires were used: the Checklist Individual Strength (CIS) [11], the Patient-Reported Experience Measure (PREM) for oncology – colorectal cancer [12], the Individual Recovery Outcome Counter (I.ROC) [13], and a Positive Health questionnaire (PH42) [14]. These instruments were selected to assess health-related well-being and patient experiences in the CRC pathway, acknowledging that formal validation in CRC populations has not been performed. Data were collected at a single timepoint.

The CIS contains 20 items scored on a 7-point Likert scale (scoring 1-7). A total score is derived by summation of all item scores. It includes four subscales: Fatigue Severity (8 items), Concentration (5 items), Motivation (4 items), and Activity (3 items) [11]. The CIS is a valid and reliable tool for the assessment of fatigue, with validated empirically derived cut-offs: fatigue severity subscale score ≥ 35 to indicate severe fatigue and a total score ≥ 76 to suggest clinically relevant fatigue [11,15]. To provide appropriate benchmarks, our results were compared with data from the Dutch ROM reference group [16].

The 23-item PREM assesses domains relevant to patients, such as trust in clinicians, clarity of information, involvement of family, and attention to patient preferences [12].

The I.ROC, originally developed for mental health, measures recovery across four domains: Home, Opportunity, People, and Empowerment (HOPE). The concept of recovery shows clear similarity to the Positive Health concept; it reflects a shift from a medical model toward a holistic, person-centered perspective. Each domain contains three items scored on a six-point Likert scale, yielding a score from 12 to 72. Higher scores indicate better well-being. Although formal validation of the I.ROC in CRC populations is lacking, Van Druten et al. [17] demonstrated its applicability in a two-factor model: wellbeing, control, network, and meaningfulness (factor 1) and health, safety, and abilities (factor 2) in a general Dutch population [18]. There are currently no widely accepted clinical thresholds for I.ROC scores for our patient population; thus, interpretation in this study was based on comparisons with data from the general Dutch population (LISS panel) [17], rather than on predefined cut-offs.

The PH42, based on the My Positive Health dialogue tool [19], consists of 42 items scored on an 11-point scale covering six dimensions: acceptance and life satisfaction, physical functioning, self-management, social roles, personal development, and cognition [17]. Higher scores indicate greater resilience, autonomy, and well-being [1]. One question per domain assesses satisfaction with overall status. There are currently no widely accepted clinical thresholds or established minimal clinically relevant differences for Positive Health scores such as the PH42. Formal validation of the PH42 in CRC populations is lacking. However, van Druten et al. [18] demonstrated acceptable construct validity of the PH42 as a measure of Positive Health domains in a general Dutch population, supporting its use for exploratory assessment of well-being in our study. Therefore, interpretation was based on comparisons with data from the general Dutch population (LISS panel) [17], rather than predefined cut-offs.

#### Qualitative data collection.

For the patients, eighteen interviews were included, two of which also involved a partner. The mean interview duration was 63 minutes (range 20–103 minutes). No repeat interviews were conducted, and field notes were made.

For the healthcare professionals, the final sample comprised 23 interviews with a mean duration of 29 minutes (range 15–49 minutes). Participants represented a diverse group involved in the CRC pathway, including five nurse practitioners, two radiotherapists, one project leader, one gastroenterologist, one secretary, one surgeon, one policy advisor, one manager, one physiotherapist, one dietician, one geriatrician, one oncologist, one pre-assessment nurse, one endoscopy nurse, one colostomy nurse, one transfer nurse, one oncology nurse, and one surgical nurse. This group composition was considered appropriate because it reflects the composition of daily practice, in which nurse practitioners are more numerous and hold a central role in coordinating the CRC care pathway.

Interviews were conducted at the patient’s home, the professional’s workplace, or via video call (due to the COVID-19 pandemic). The interview guides included implicit questions about service modularity (components, modules, and interfaces), as well as experiences and perspectives on shared decision-making, person-centered care, improvement suggestions, and information provision (see S1 File). Data collection continued until thematic saturation was reached, which we defined as the point at which consecutive interviews no longer yielded novel codes or conceptual insights. Saturation was assessed through iterative comparison of emergent codes across interviews and confirmed when two subsequent interviews produced no additional themes. Member checking was not performed.

### Data analysis

The data consisted of transcripts of the interviews, the completed questionnaires, and conceptual memos based on the available documentation. The different types of data were complementary to each other: the questionnaires helped to acquire information on the patient characteristics and disease-related symptoms, the interviews helped to acquire information on the professional’s and patient’s perspective on care provision, and the collected documents gave valuable information about the organization of CRC care in the JBZ.

#### The lens of service modularity.

Service modularity refers to the design and organization of service processes into distinct, standardized, interchangeable modules, each comprising a set of components to meet individual needs [20]. Components represent the smallest elements into which a service can be meaningfully decomposed [21]. Modules can be flexibly configured into tailored packages without loss of functionality, with interfaces managing communication between them to ensure compatibility [20]. Such a modular structure enhances flexibility, efficiency, and customization [21]. In healthcare, it enables the decomposition of care pathways into hierarchical structures, where components can be adapted or replaced without affecting the overall system.

We collected additional data on the CRC pathway at the JBZ to map its service architecture, including the CRC protocols. These protocols are based on several guidelines and quality criteria from organizations involved in oncological care: 1) Quality criteria of the Dutch Federation of Cancer patient organizations, 2) guideline colorectal carcinoma 2014, version 3, 3) DCSA Dutch Surgical Colorectal Audit, 4) Pathway for cancer patients needs improvement, 5) Pallialine, Palliative indicators, 2010), 6) Dutch working group Gastro intestinal tumors, SONCOS norms (November 2013), 7) Dutch guideline coloscopy surveillance, and 8) the EMBRAZE cancer network.

By synthesizing interviews and documentation from a modularity perspective, we analyzed whether and how healthcare professionals and patients enacted and experienced care from a modularity perspective. We used modularity as an analytical framework to interpret the data, whether or not participants were familiar with the concept of modularity or explicitly refer to it.

#### Quantitative analysis.

We analyzed the data using IBM SPSS Statistics for Windows (Version 27). Descriptive statistics were used to describe the results of the questionnaires of patients involved in the CRC pathway. Missing data was handled by excluding the participant in the analyses concerning the specific question(s) in which this data was missing.

#### Qualitative analysis.

Interviews were audio recorded, transcribed verbatim, and analyzed using ATLAS.ti (Version 2023). We adopted an abductive thematic analysis approach, combining deductive and inductive coding methods [22]. The analysis started from a predefined coding scheme informed by literature and theoretical frameworks. The framework of McCormack et al. [23] guided the coding of person-centered care and shared decision-making themes. In parallel, inductive coding identified new and unexpected patterns. This abductive strategy enabled iterative movement between data and theory, refining our understanding of the phenomenon. New codes were added as they emerged, and the coding scheme was continuously updated to reflect both pre-determined and emergent themes. This approach ensured theoretical grounding while remaining open to novel insights.

Separate coding schemes for patients and healthcare professionals were drafted after familiarization with the data and existing literature. These preliminary coding schemes were used by all researchers to code the first interview with healthcare professionals and the first two interviews with patients. The coding schemes were iteratively refined through team discussions until final agreement was achieved (see S2 Table). All interviews were then independently coded by two researchers (JT and MK for patient interviews, BM and MK for healthcare professional interviews), followed by regular discussions to resolve discrepancies and ensure consistent interpretation. Themes were prioritized based on the depth and significance of the codes, and preliminary themes were reviewed in team meetings to refine and consolidate them. Related codes were clustered and integrated into broader overarching themes, supporting a coherent thematic structure.

### Ethical considerations

Ethical approval for this study was obtained from the Ethics Review Board of Tilburg University [EC-2019.61]. Written informed consent was obtained prior to participation from all participants.

## Results

### Modular perspective

**Fig 1:**
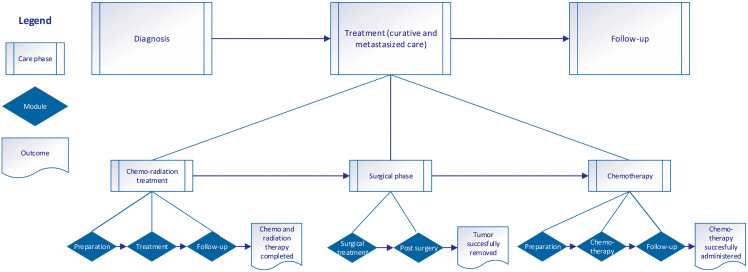
Overview of multilevel process and outcome-oriented decomposition logic for the current CCP.

The CRC pathway protocols provide the foundation for visualizing the current service architecture. The existing decomposition logic (referring to how the care process is broken down into smaller, manageable components) integrates both outcome- and process-oriented approaches within a multilevel hierarchical structure. At the first level, the pathway is divided into three primary phases: diagnosis, treatment, and follow-up. At the second level, each phase is decomposed into modules, which comprise interconnected components with defined interfaces. Each phase is designed to achieve a distinct specific outcome, such as accurate diagnosis or successful surgical intervention. Within each phase, modules are structured such that the output of one module serves as input for the next module, ensuring a seamless transition across the treatment trajectory. This framework is visually represented in Fig 1.

First, the diagnosis phase comprises four clinical encounters, beginning with the initial colonoscopy intake and ending with a surgical consultation, where the definitive treatment plan is established. Second, the treatment phase is structured into three key modules: 1) treatment preparation, 2) the treatment itself, including chemoradiation and chemotherapy for patients with lymph node-positive CRC, and surgical intervention, and 3) treatment evaluation. Last, the follow-up phase encompasses a structured monitoring scheme, incorporating various components aimed at supporting long-term patient care and early detection of recurrence. Each module contains multiple interrelated components, ensuring continuity of care. A more detailed visualization of the current CRC pathway is provided in S3 Fig.

## Patients with colorectal cancer

### Questionnaires

#### Study participants.

Forty-three percent of the patients were female; the median age was 72 (IQR 19; min-max 45-87). Eighty-five percent of the patients was in the follow-up phase of treatment; 53% of the patients had a low educational level. Further baseline characteristics (derived from the PREM questionnaire) are shown in Table 1.

**Table 1:** paBaseline characteristics of the patients who completed the questionnaires.

Characteristic	Patients with colorectal cancer n = 65
**Sex**, n (%)	
Female	28 (43)
**Age**, median (IQR; min-max)	72 (19; 45-87)
**Phase of treatment**, n (%)	
Diagnostic phase	2 (3)
Curative treatment	5 (8)
Follow-up	55 (85)
Treatment and follow-up are	3 (5)
completed	
**Education level**, n (%)	n = 64
Low	34 (53)
Middle	16 (25)
High	14 (22)

**IQR** Interquartile range; **min** minimum; **max** maximum; **Educational level. Low:** primary education, prevocational secondary education, first three years of senior general secondary education, first three years of pre-university education and senior secondary vocational education level 1. **Middle**: four or five years of senior general secondary education, four, five or six years of pre-university education and senior secondary vocational education, level 2, 3 or 4. **High**: higher vocational education and university education.

#### Health-related wellbeing.

Table 2 provides an overview of the baseline outcome measurements of the CIS, I.ROC, PH42 questionnaires, and general population values as a reference. In the subsequent section (see ‘psychosocial impact of CRC treatment’), findings from the interviews and the questionnaires will be presented in an integrated manner.

**Table 2:** Baseline outcome measurements of the CIS, I.ROC and PH42 questionnaires.

	Patients with colorectal cancer n = 65	ROM reference group [16] n = 643
**Checklist Individual Strength**, median sum scores (IQR; min-max)		
Fatigue severity (scale 8–56)	37 (8; 24–47) n = 63	16 (18; 8-46)
Total sum score (fatigue, concentration, motivation,activity) (scale 20–140)	84 (10; 57–97) n = 63	38 (32; 20-140)
	**Patients with colorectal cancer** n = 65	**General Dutch population, LISS panel [17]** n = 2457
**I.ROC**, median sum scores per factor (IQR; min-max)		
Wellbeing, control, network, and meaningfulness (scale 8–48)	37 (8; 23–47) n = 58	36 (9; 10-48)
Health, safety, and abilities (scale 4–24)	20 (4; 9–24) n = 58	20 (4; 7-24)
Total score (scale 12–72)	58 (12; 34–69) n = 58	56 (11; 21-72)
**PH42**, median sum score per factor (IQR; min-max)		
Acceptance, meaningfulness and satisfaction with life (scale 0–130)	110 (26;54–130) n = 61	102 (25; 9-130)
Physical health and functioning (scale 0–80)	59 (23; 21–80) n = 59	60 (16; 7-80)
Self-management (scale 0–70)	63 (14; 35–70) n = 61	59 (11; 18-70)
Social network and societal roles (scale 0–80)	70 (12; 40–80) n = 61	64 (14; 4-80)
Personal development (scale 0–30)	25 (5; 12–30) n = 62	22 (6; 3-30)
Cognition (scale 0–30)	25 (5; 7–30) n = 65	24 (6; 2-30)
Total score (scale 0–420)	348 (72; 182–420) n = 57	330 (66; 104-420)

**CIS** Checklist Individual Strength; **IQR** Interquartile range; **min** minimum; **max** maximum; **I.ROC** Individual Recovery Outcome Counter; **LISS** Longitudinal Internet Studies for the Social sciences; **PH42** My Positive Health Questionnaire (42 items); **ROM** Routine Outcome Measuring.

### Interviews

#### Study participants.

In total, 21 patients provided consent for an interview; more detailed information about the interview procedure is provided in the Methods section.

Several overarching themes and associated code clusters reflecting patients’ experiences throughout the care pathway were identified in the interviews. An overview of these themes is provided in Table 3.

**Table 3:** Overview of themes, codes, and exemplar quotes from the interviews with patients.

Theme	Code clusters	Examplar quotation
**Aspects of person-centered care**	Involvement of healthcare professionals, approach to patients, trust in healthcare professionals	“The support that the doctor gave to my wife and me was 1000%.” (P11) “… Those people were all so, it must be the ward, but they were all so compassionate.” (P3)
	Feeling heard en taken seriously	“I could say what I wanted, …, and ask whatever I needed.” (P15)
**Patient involvement in shared decision making**	Trust in treatment, trust in healthcare professional, proactive care planning, quality of life, perceived choice in treatment decisions	“No, I did not have a choice. The only option was surgery.” (P5)
	Preference for guidance versus autonomy	“What I really appreciated was that they [healthcare professionals] truly let you make the choice yourself...” (P18)
**Psychosocial impact of CRC treatment**	Coping, resilience, mental care, involvement of relatives, psychological support	“… Well, I chose to do everything I could to get better.” (P3)
	Fatigue, emotional impact	“In the evenings, I am just tired, really tired.” (P6)
	Fear of recurrence, uncertainty	“But I actually found that these follow-up check-ups had more impact on me than the first time.” (P2)
**Perspective on the role of the nurse practitioner and communication**	Nurse practitioner as a human connection element, communication with the general practitioner	“… At least, I experienced it as very pleasant (P4) “The discharge letter did reach the general practitioner, but it was incomplete. The full detailed report arrived weeks later.” (P5)
**A need for tailored information and digital tools**	Information overload, written summaries, visual aids	“…You hear it all during the consultation but you forget half of it. At home you can read it again whenever it suits you. And yes, that was ok for me.” (P4)
	Use and limits of digital tools and patient portal	“The digital patient portal is very convenient, but it is a pity there is no medical glossary...” (P12)
**Experienced effects of prehabilitation**	Peer support, appreciation of the prehabilitation program, nutritional guidance	“… But at that moment, I just did not see the point of it [prehabilitation program].” (P8) “I enjoyed it [prehabilitation program], and you meet people who are deadline with the same issue. That is nice.” (P7)
**Patient-identified opportunities to improve care coordination**	Appointment clustering, waiting times, patient autonomy in appointment planning	“I would have appreciated it if they had asked me: would you like to receive the information, the results, as soon as possible? Personally, they are welcome to call me with either good or bad news, as soon as they know something.” (P6)

### Aspects of person-centered care

Patients were generally satisfied with professional involvement; doctors and nurses engaged regularly, including family, and aimed to reduce waiting times. Patients appreciated having a voice in their care trajectory, with their personal preferences and situations considered. One patient noted the need for more attention to the human side of the doctor-patient relationship.

*- “The support that the doctor gave to my wife and me was 1000%.” (P11)*

*- “Whether it was a lady from catering bringing breakfast in the morning, or the man or woman doing the blood draws, those people were all so… it must be the ward, but they were all so compassionate.” (P3)*

Patients appreciated professionalism, direct and approachable communication, and individualized care from healthcare professionals. Most felt heard and taken seriously, though one reported feeling dismissed. Some noted unclear answers or disengaged attitudes. Overall, care quality was perceived as high, with nurses managing discomfort effectively. Bad news was communicated professionally. However, some areas for improvement were identified, including paying more attention to a patient’s discomfort during a procedure and improving the accuracy of the provided information.

*- “Well, that [bad news] was tough of course, but it was communicated very well, very... I cannot say pleasant, but definitely professional.” (P12)*

*- “I cannot say otherwise, I can really only be positive about how they treated me, and how I was able to interact with them [healthcare professionals]. I could say what I wanted, yes, politely of course, and ask whatever I needed.” (P15)*

### Patient involvement in shared decision-making

Patients generally trusted their healthcare professionals and felt that treatment options were appropriate. However, some did not feel they truly had a choice, even when options were presented. While many valued making informed decisions to avoid unnecessary invasive treatments, others preferred to just follow professional advice. One patient declined surgery, which led to team review, but still felt openness to alternatives.

*- “And I believe that is how it goes every time, that those people come together and say, well, we are going to do it this way and that way. All to get the best possible outcome for the patient.” (P1)*

*- “No, I did not have a choice. The only option was surgery.” (P5)*

Patients generally felt involved in chemotherapy decisions, guided by quality of life. One patient felt that their choice was not respected, as chemotherapy seemed predetermined. Risks, benefits, and success rates were clearly explained, and patients valued open discussion, including about unproven treatments such as hyperthermia.

*- “The choice was not given to me. No, the oncologist immediately said, “You can start chemotherapy in 14 days… or next week.” And I said, “I would still like to take some time to think about it first.” (P5),*

*- “What I really appreciated was that they [healthcare professionals] truly let you make the choice yourself. They would say, “These are the benefits of X, and these are the benefits of Y.” (P18),*

### Psychosocial impact of CRC treatment

Patients varied in their mental approach. Some were passive, others sought control through information. Coping levels varied among patients, with some reporting little psychological impact and other experiencing stress-related symptoms like tension and fatigue.

*- “Look, I knew I had cancer. Then you have got two options: you can take part in everything, even if it only helps a little bit, or you can stay at home. Well, I chose to do everything I could to get better.” (P3)*

*- “I just felt perfectly fine.” (P1)*

*- “In the evenings, I am just tired, really tired.” (P6)*

*- “I do know that I was not calm [right after surgery]. They told me later that they had to calm me down. That is when I thought: oh, so I was afraid, but I had been suppressing it. And now it had actually come to the surface.” (P7)*

**Fig 2:**
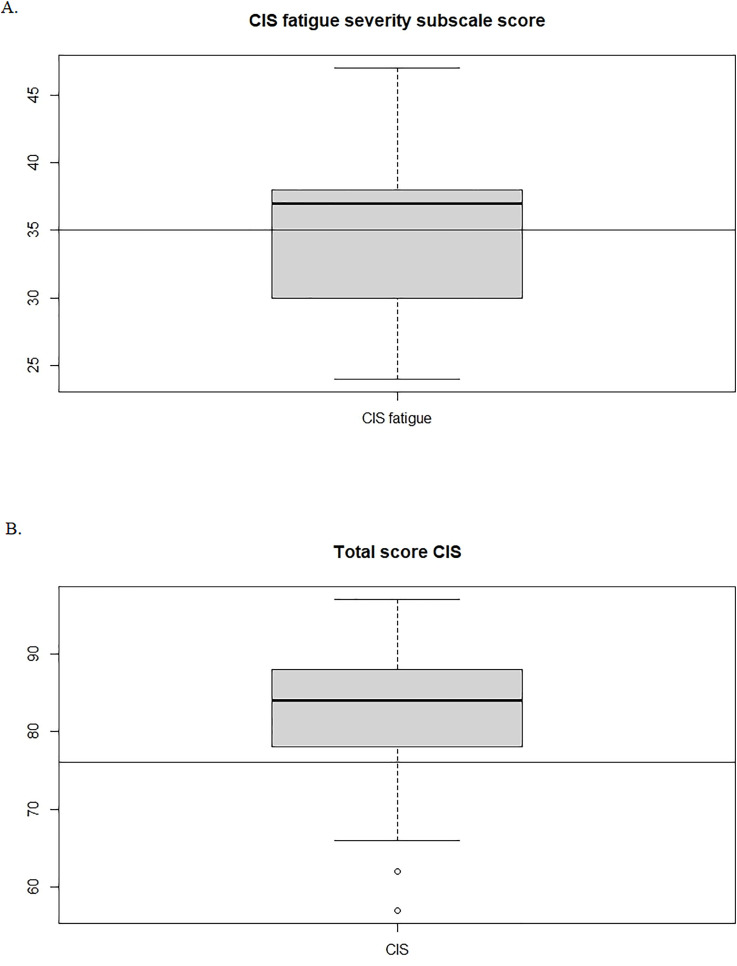
A. Boxplot of CIS fatigue severity subscale scores, and B. total score CIS in the study group. The horizontal line indicates the cut-offs points for severe and clinically relevant fatigue (fatigue severity = 35 and total CIS score = 76).

This variation in experienced fatigue was reflected in the CIS fatigue severity subscale scores of the 63 patients who completed the questionnaire. Patients had a median score of 37 (IQR 8; range 24–47), compared with a median of 16 (IQR 18; range 8-46) in the reference group, indicating high fatigue levels (see Table 2). The median total CIS score was 84 (IQR 10; range 57-97) compared with 38 (IQR 32; range 20-140) in the reference group, also indicating clinically relevant fatigue. The distribution of fatigue severity subscale scores and total CIS scores of our study are shown in Fig 2A-B.

Most patients saw the treatment as something to endure, with no real alternative. Many experienced anxiety during waiting periods, especially in follow-up due to fear of reoccurrence. To reduce this anxiety, some patients often preferred more frequent follow-up tests, which provides a sense of reassurance. Some also worried about postoperative complications.

*- “But I actually found that these follow-up check-ups had more impact on me than the first time.” (P2)*

Patients generally felt that mental health support was sufficient and perceived no need for routine psychological screening. Social support from family and friends was seen as crucial for emotional stability and coping.

*- “I spend a lot of time with my partner, and we have a wonderful daughter. She was really my support. I could talk to her about everything, and we did talk a lot. There was definitely space for that, yes. They [healthcare professionals] also offered it, saying if you want to talk about it, you can always call.” (P9)*

The questionnaire data showed that the median PH42 score was higher in CRC patients than in the reference group (348 vs. 330), also reflecting the stabilizing effect of strong social networks. Similarly, the I.ROC scores suggest relatively favorable self-perceived recovery (median 58; IQR 12; range 34–69), slightly exceeding the reference group (median 56; IQR 11; range 21–72). A detailed overview of the quantitative results is provided in Table 2.

### Perspective on the role of the nurse practitioner and communication throughout the care pathway

Patients found the nurse practitioner highly valuable, citing their approachability and the development of an emotional bond through long-term, multidisciplinary contact. The nurse practitioner served as the primary point of contact, ensuring that patients receive continuous care and support throughout their treatment.

*- “I assume that someone is hired dedicated for this [tasks nurse practitioner], and that is very pleasant. At least, I experienced it as very pleasant.” (P4)*

Patients perceived sufficient coordination among specialists but noted gaps in communication during hospital stays. The communication with the general practitioner was often poor, with delayed or incorrect information and missing test results.

*- “The discharge letter did reach the general practitioner, but it was incomplete. The full detailed report arrived weeks later.” (P5)*

### A need for tailored information and digital tools

Patients generally felt well-informed, which supported their understanding of the care pathway. The initial information load was sometimes overwhelming. Written summaries and visual aids were appreciated, as was the ability to review information at their own pace. Information folders were used variably but seen as clear and follow-up schedules provided structure and clarity.

*- “I received the information [from the nurse practitioner] in person and printed in the*

*information folder through which I can read it all again later at home. You hear it all during*

*the consultation but you forget half of it. At home you can read it again whenever it suits*

*you. And yes, that was ok for me.” (P4)*

*- “So, the nurse practitioner gave me a kind of schedule, an overview of what will happen over the next five years. I find it reassuring, it helps to know what to expect.” (P4)*

Most patients used the digital portal to view test results in advance, which helped them prepare for appointments. A suggested improvement was the inclusion of a glossary within the portal to explain medical abbreviations and terms. One patient worried that digitalization might reduce personal contact. Opinions on videos were mixed, though some determined value for those with language or reading difficulties.

*- “The digital patient portal is very convenient, but it is a pity there is no medical glossary. For example, in a blood test or lab report, reference values are shown, but only abbreviations are used. You want to know what they mean.” (P12)*

*- “I can imagine that the information sticks better if you can visualize it, especially for people who are older or who do not have a strong command of the language. That could work really well.” (P5)*

### Experienced effects of prehabilitation program in the preoperative phase

Patients generally valued prehabilitation, though some questioned its relevance if they felt fit. Earlier communication was suggested to improve preparedness. Experiences varied, but dietitian support was seen as helpful for lasting dietary changes. Peer support was appreciated for both practical and emotional connection. Furthermore, postoperative home support varied. Some felt well supported, others reported limited access to physiotherapy and rehabilitation.

*- “I thought, I am about to start that therapy [prehabilitation program] and I will not get anything out of it. The surgeon said, yes, but those are different exercises. You will never be worse off from it. I do not doubt that, but at that moment, I just did not see the point of it.” (P8)*

*- “I enjoyed it [prehabilitation program], and you meet people who are dealing with the same issue. That is nice.” (P7)*

### Patient-identified opportunities to improve care coordination

Patients described mixed preferences regarding appointment scheduling. Some wished to combine appointments across specialties, though this was not possible within the current system. They also expressed interest in having a choice between in-person and virtual consultation. While support scheduling was seen as adequate, the waiting time between tests and results was often experienced as long. Communicating results earlier, for instance by phone, was suggested as a useful improvement.

*- “I would have appreciated it if they had asked me: would you like to receive the information, the results, as soon as possible? Personally, they are welcome to call me with either good or bad news, as soon as they know something.” (P6)*

### Healthcare professionals involved in the CRC pathway

The interviews with healthcare professionals resulted in several overarching themes and associated code clusters that capture their perspectives on the organization and delivery of care. A detailed overview is provided in Table 4.

**Table 4:** Overview of themes, codes, and exemplar quotes from the interviews with healthcare professionals.

Theme	Code clusters	Exemplar quotation
**Integration of person-centered care in clinical practice**	Language use, approach to patients, involvement of relatives, accessibility, expectation management, doctor-patient relationship	“… We want to provide good care for someone, and for that, the treatment relationship has to be optimal.” (H7)
**Integrating quality of life in person-centered care**	Life goals, treatment success, advanced care planning, focus on physical and mental health status.	“…We always begin with the key question: what can we do for you, and what would make this consultation meaningful?” (H15) “… and especially with palliative patients, I ask: how is your quality of life? How do you experience it, and what is important to you?” (H1)
	Lack of structural follow-up, social and work-related challenges	“… But currently, there is no structural support for that [rebuild their lives.” (H8)
**Shared decision-making in treatment options**	Information provision, perceived choice, protocol-driven decisions	“The patient always has a choice… As long as they understand the pros and cons…” (H1)
	Curative dominance, age assumptions	“… I think there are very few reasons not to offer a treatment to a young, fit patient … So yes, I do pay attention to it, but mainly for that selected group of frail older patients” (H5)
	Duty of care, discussing alternatives	“Some doctors experience a barrier to proposing an alternative when the patient already indicates a different treatment preference…” (H7)
**The role of nurse practitioners in interdisciplinary care**	Continuity, accessibility, reassurance, signaling function	“What we do very well, I think, is having a cross-disciplinary nurse practitioner. This means that patients essentially have one consistent point of contact throughout the entire process …” (H7)
	Time availability, psychosocial awareness	“We [nurse practitioners] have considerable time to reflect on who these patients are and how they are doing.” (H18)
**Enhancing collaboration in the care pathway**	Cross-disciplinary work, communication channels	“It would be ideal to have a shared EHR. That is really a missing element. The collaboration with radiotherapy center runs smoothly, but there is no feedback loop.” (H1)
	Fragmented EHRs, duplication, lack of feedback loops	“… That is an area where efficiency could definitely be improved.” (H18)
**Perspectives on patient information provision**	Method of information delivery, amount of information, content of information	“… They [patients] are faced with another flood of information.” (H9)
	Use of apps, digital patient portal	“… Right now, there is an overgrowth of apps and digital tools.” (H3)
**Perspectives on the prehabilitation program**	Content of the prehabilitation program, importance of attention to physical functioning, appreciation of the prehabilitation program, peer support	“… In those [patient] cases, they prefer to take two extra weeks for training, because that can really make a significant difference.” (H11)
**Suggestions to enhance the care process**	Standard procedures, capacity constraints, scheduling limits, hospital logistics, limited flexibility	“It would be ideal to schedule as much as possible on a single day, but that is becoming increasingly difficult. This is partly due to the fact that we now have more medical specializations.” (H9)

### Integration of person-centered care in clinical practice

Healthcare professionals generally felt that person-centered care was well embedded in daily practice. The care pathway was seen as structured yet flexible, with extra appointments arranged when needed. Some professionals saw room for improvement in scheduling, information provision, and outpatient visit planning. Individualized agreements were not consistently recorded in the EHR, and one professional noted that care was often tailored to the disease rather than the person. Healthcare professionals stressed that customization must remain medically justified.

*- “But if I know the patient, we give the oncological recommendation, but we always try to offer a tailored alternative as well.” (H7)*

*- “Perhaps patients could have even more control over how things are managed. At the moment, we are quite directive in how we organize care. Of course, we do involve the patient in many decisions, but perhaps still not enough.” (H9)*

To ensure optimal care, professionals emphasized the importance of a strong patient-professional relationship. When this was lacking, a patient could change professionals or hospitals. Professionals noted that patients could be more actively involved and that surgical decisions should better reflect individual needs. They highlighted the need for greater informal caregiver involvement.

*- “Some patients have had bad experiences in this hospital, and then I say: “if you do not have that trust, I can never fully restore it... We want to provide good care for someone, and for that, the treatment relationship has to be optimal.” (H7)*

### Integrating quality of life in person-centered care

Healthcare professionals consistently addressed quality of life during consultations, and they discussed personal goals and treatment success with the patient early in the pathway. Advanced care planning documented preferences and aided decision, however, one professional noted its current focus on palliative care and suggested broader use for curative patients. Furthermore, they observed that patients focused more on physical than mental health, but still felt mental well-being being adequately addressed. Both aspects were integrated into care, with mental health discussed more often in palliative patients. Family members were frequently involved in these conversations.

*- “I always ask patients what they want to achieve, what would make the surgery successful, and what their life goals are. For some, it is prolonging life, for others, quality of life. We always begin with the key question: what can we do for you, and what would make this consultation meaningful?” (H15)*

*- “I examine both physical and psychological aspects of the patient, and especially with palliative patients, I ask: how is your quality of life? How do you experience it, and what is important to you?” (H1)*

Time constraints limited attention to patients’ full well-being. Professionals acknowledged the need to address concerns during consultations and referred patients to services within the institution when possible. They recognized the need for more support in social, emotional, financial, and work-related areas. Efforts were made to establish structured collaborations with regional partners to screen patients for potential challenges and provide comprehensive support beyond medical care, including work-related concerns and family involvement.

*- “You notice that after treatment there is a significant gap, patients do not know how to rebuild their lives. These questions often arise after some time. For some, it may be within two weeks; for others, it might take months or even years. But currently, there is no structural support for that.” (H8)*

### Shared decision-making in treatment options

Healthcare professionals emphasized the importance of informing patients for decision-making and felt this was achieved in practice. In palliative care, some used a web-based advanced care program and experienced more flexibility to deviate from standard protocols. For patients eligible for curative treatment, some professionals discussed alternatives less often, assuming curative care was preferred. Others stressed the need to confirm patients’ wishes regardless of medical eligibility. While professionals recognized the right to decline optimal treatment, some found this difficult to accept.

*- “The patient always has a choice. I believe we must ensure the patient is well informed so they can make a well-considered decision, whether the treatment is adjuvant or palliative. Even if people choose an alternative approach, that is their choice. As long as they understand the pros and cons, enabling them to make an informed decision.” (H1)*

*- “It is more about the disease, except when it is really an older, frail patient. Then I find what truly matters to people in their lives much more relevant. Because at that point, you really have to determine whether it is appropriate to subject someone to an oncological treatment. And I think that for younger, fit patients, it is not as relevant at that moment…. I think there are very few reasons not to offer a treatment to a young, fit patient … So yes, I do pay attention to it, but mainly for that selected group of frail older patients.” (H5)*

*- “Some doctors experience a barrier to proposing an alternative when the patient already indicates a different treatment preference. However, Dutch law and the duty of care doctors have provided ample room for this. In fact, some people have been convicted for blindly following the multidisciplinary team’s advice, even though the patient later said another treatment was available and was not discussed with them. Disciplinary boards have ruled that doctors should have discussed this because it is part of their professional responsibility.” (H7)*

Healthcare professionals based treatment decisions on quality of life and overall health, sometimes opting for less or no treatment. While professionals could provide strong recommendations, these were not mandatory. The multidisciplinary team proposed a treatment plan as a guideline, not a directive and in-person consultations were seen as essential to determine the best approach.

*- “Well, I outline the pros and cons and provide advice to the patient. Some patients say, doctor, you just tell me what to do. So, what I do is ask patients what they find important in life and how they approach life. I say, you can tell me that, and based on it, I can give you an advice.” (H7)*

### The role of nurse practitioners in interdisciplinary care

Healthcare professionals noted that the nurse practitioners interdisciplinary and proactive role provides patients a single contact point throughout treatment. This role was highly valued as the nurse practitioner linked professionals and enhanced person-centered care. Extended contact time also allowed the nurse practitioner to monitor patients’ mental health and well-being effectively.

*- “What we do very well, I think, is having a cross-disciplinary nurse practitioner. This means that patients essentially have one consistent point of contact throughout the entire process. That is very reassuring for patients.” (H7)*

*- “I do think that a lot of care is tailored to the individual because patients have a central point of contact in the form of the nurse practitioner.” (H10)*

*- “As nurse practitioners, I believe we play an important role. We have considerable time to reflect on who these patients are and how they are doing.” (H18)*

### Enhancing collaboration in the care pathway

Healthcare professionals generally viewed collaboration as effective, supported by the nurse practitioners cross-disciplinary role and consistent documentation in the EHR. Communication with other specialties occurred through various channels and was seen as adequate. However, professionals called for better pathway integration, improved ward handovers, and shared access to radiotherapy data via a shared EHR.

*- “We [nurse practitioners and doctors] work in the medical record, while the ward nurses use the nursing record. Much of the patient information is already in the medical record, but nurses have to enter it again in the nursing record. That is an area where efficiency could definitely be improved.” (H18)*

*- “It would be ideal to have a shared EHR. That is really a missing element. The collaboration with radiotherapy center runs smoothly, but there is no feedback loop.” (H1)*

### Perspectives on patient information provision

Healthcare professionals observed that despite providing comprehensive information, patients often retained limited details due to the volume presented. One professional suggested a ‘menu’ allowing patients to choose relevant information at their own pace while ensuring essential content was conveyed. Professionals adapted their language and approach to patients’ understanding but found it challenging to assess comprehension in patients with low literacy, as needs were not systematically evaluated early in the care pathway.

*- “Well, the amount of information is quite a lot. They have just received the diagnosis, and then they are immediately told what will happen next and why. It is overwhelming for them, because they have only just heard what is going on, and then they are faced with another flood of information.” (H9)*

*- “You have highly educated patients who ask many questions and are very rational. On the other hand, there are those who are guided more by emotion and feel very anxious. I adjust to that.” (H1)*

Healthcare professionals noted that visual tools such as apps could support information delivery but stressed that personal conversations remained essential, especially for discussing test results. They proposed phased information aligned with treatment stages but raised concerns about fragmentation due to the abundance of apps. Professionals also called for more tailored information and better documentation in the EHR.

*- “I would prefer a single platform where all specialties can contribute information. That way, the patient has one place to access everything. Right now, there is an overgrowth of apps and digital tools.” (H3)*

Professionals noted active patient use of the digital portal to access results and complete questionnaires. Although useful, the portal posed challenges when patients viewed results before consultation, sometimes causing distress. Experiences with e-consultations were mixed. While seen as part of future care, the current system lacked infrastructure and time allocation. Professionals also highlighted the need for clearer guidelines on when in-person versus remote communication is appropriate.

*- “You do notice it in the consultation room. Some people already know their results, and others do not.” (H5)*

### Perspectives on the prehabilitation program

Healthcare professionals emphasized that preoperative fitness is key to better surgical outcomes. A prehabilitation program was implemented to strengthen frail patients, with surgical eligibility assessment sometimes postponed to after prehabilitation. Professionals observed both physical and psychological benefits, including therapeutic peer support through group training and greater patient engagement in the treatment process.

*- “Some patients do not yet have a set date for surgery. The surgeon says: let’s focus on training first, and we will decide later when the operation would be most appropriate … In those cases, they prefer to take two extra weeks for training, because that can really make a significant difference.” (H11)*

### Suggestions from professionals to enhance the care process

Healthcare professionals generally regarded the care pathway effective but identified areas for improvement. Some procedures, like routine restaging, were seen as unnecessary but remained standard. Dependence on departments such as radiology limited efficiency and patient control over scheduling, which was driven by hospital capacity. Despite these constraints, efforts were made to accommodate patient preferences as much as possible.

*- “I truly think this is the best-functioning care pathway we have.” (H5)*

*- “It would be ideal to schedule as much as possible on a single day, but that is becoming increasingly difficult. This is partly due to the fact that we now have more medical specializations.” (H9)*

## Discussion

We applied the composite model developed by Bartels et al. [7] as a conceptual ground to study current practice within the JBZ CRC pathway. Our study had two goals: 1) to explore the applicability of the composite model in a real-world case as a form of theory-testing, and 2) to evaluate how person-centered care, shared decision-making, and service modularity are perceived and enacted by both patients and healthcare professionals in the JBZ CRC pathway. By projecting the model onto current practice, we aimed to identify areas for improvement and to generate insights into how modular service design can better support meaningful, customized cancer care.

**Fig 3:**
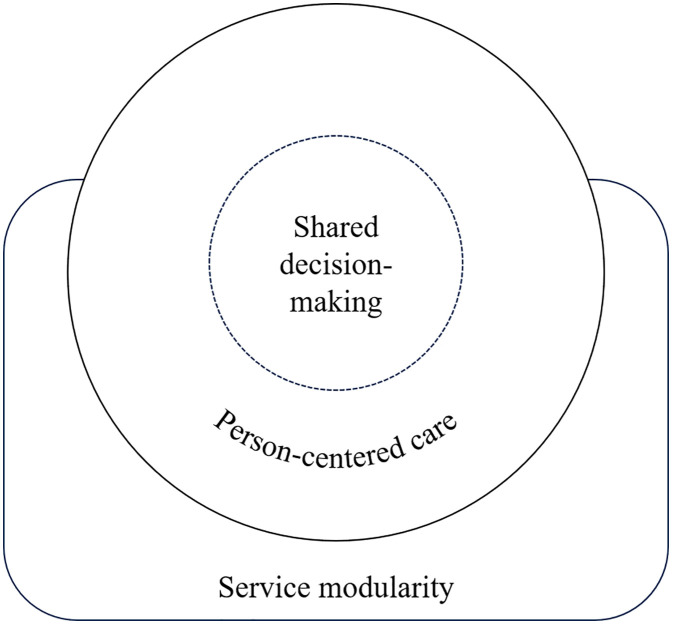
Elaboration of the composite theoretical model, illustrating the central proposition of Bartels et al. [7]: “*the potential of service modularity as a foundation for person-centered care delivered in a shared decision-making context*”.

**Fig 4:**
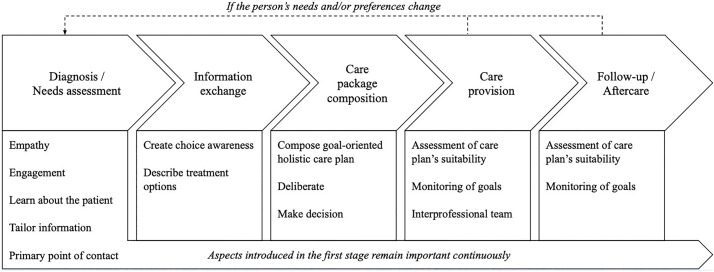
Person-Centered Service Specification Process in a Shared Decision-Making Context (source: Bartels et al. [7]).

To address the first aim, we applied the model as an analytical lens to structure our case study analysis. The model proved conceptually robust and practically valuable, revealing misalignments between formal care structures and actual care. For example, the model revealed information breakdowns between hospital teams and general practitioners during discharge, exposing gaps in care coordination that formal structures did not address. Findings illustrated how person-centered care, shared decision-making, and service modularity are enacted in the JBZ CRC pathway. For instance, limited modular thinking among professionals can hinder customization, as care packages were routinely offered without exploring patient preferences. Fig 3 visualized these findings, which builds upon the original conceptual representation (Fig 4) by illustrating how the model’s three components enacted in practice.

Thus, the model contributed both to the theoretical validation of the composite framework of Bartels et al. [7] and to practical insights in the JBZ CRC pathway. Specifically, it illuminated aspects that might have remained unnoticed when focusing on the model’s theoretical aspect only. The following sections deepen the analysis of the second aim across the phases shown in Fig 4.

### Diagnosis/needs assessment

Information provision is essential for patient engagement, comprehension, and informed decision-making throughout the care pathway [24]. In the JBZ CRC pathway, the structured protocol ensures completeness but limits flexibility, as it insufficiently accounts for individual preferences regarding timing and volume of information. This can lead to cognitive overload and reduced engagement [25]. Unlike prior studies focusing solely on communication or logistics [26], our approach shows the need for flexible, patient-tailored strategies, such as stepwise delivery supported by digital tools. These approaches promote patient autonomy and comprehension while minimizing informational overload.

Patients emphasized the importance of empathy, engagement, and being known as a person. These qualities are embodied in the nurse practitioner as primary point of contact in the pathway. The nurse practitioners cross-disciplinary role ensures continuity and connects different care modules, extending beyond the commonly described relational or operational terms [26].

### Information exchange

Information exchange aligns clinical expertise with patients values through open communication, creating choice awareness, and presenting treatment options. Although the JBZ CRC pathway supports structured communication, our analysis showed that preference elicitation is not systematically embedded. Especially in curative trajectories, treatment decisions often rely on implicit assumptions, such as a presumed preference for curative intent, rather than explicit exploration which may limit patient autonomy [27]. Integrating structured methods to identify and incorporate preferences would support *truly* shared decision-making. In a separate study, Peters et al. [28] noted limited attention to interfaces that support direct and indirect interaction, such as needs assessments or information letters.

### Care package composition

To foster patient involvement within clinical constraints, professionals could adopt a more modular approach to care composition, translating patient preferences into more flexible care [7]. Viewing care as adjustable components allows preferences to be better integrated without compromising clinical appropriateness. Our analysis showed that even when elements of shared decision-making are present, care often reflects routine practice more than individual priorities. Option framing often mirrors professional preferences, potentially constraining the perception of autonomous patient choice.

Customization is more challenging when curative options are constrained by tumor characteristics or metastases [29]. Professionals may have difficulty to align care with patient preferences, especially when these diverge from medical recommendations. Yet shared decision-making also involves space for patients to voice values that may contradict clinical advice. Our findings show that professionals aim to create room for this by tailoring their interpersonal approach, though some find it difficult when patients decline clinically preferred treatments. A modular setup helps focus on the consequences of flexibility within the clinical context [7].

### Care provision

Care transitions in the JBZ CRC pathway were generally well-coordinated, supported by the nurse practitioners cross-disciplinary role. However, communication lapses during hospitalization revealed vulnerabilities in inpatient transitions, highlighting the need for clearer roles and stronger leadership in multidisciplinary teams.

Prehabilitation responses varied: many patients appreciated the program, others questioned its personal relevance. Aligning services with perceived patient value is important [30], as is offering autonomy in scheduling and consultation formats. These preferences point to opportunities for enhancing person-centeredness by offering more flexible and integrated appointment structures [31]. Increasing flexibility in these areas could enhance patient-centeredness and continuity, especially during transitions involving multiple professionals or care settings.

### Follow-up/aftercare

Fatigue emerged as a major issue during treatment and follow-up, with questionnaire data indicating clinically relevant fatigue (median total CIS score 84, fatigue threshold total CIS score ≥ 76 [15]). The CIS fatigue severity subscale scores also indicated severe fatigue with a median score of 37 (threshold subscale score ≥ 35 [11]). This aligns with literature describing fatigue as a common, persistent symptom in cancer patients [32]. Interestingly, in our study patients rarely mentioned it unprompted in the interviews, while professionals routinely addressed it in consultations. This disconnect may reflect that responses on standardized questionnaires do not always correspond directly to patients’ lived experiences or the issues they perceived as most pressing [33]. Patients may normalize fatigue, focus on other aspects of daily functioning, or consider it an expected consequence of treatment. These findings may underline that questionnaires, while valuable, cannot fully replace in-depth conversations with patients [33]. In addition to fatigue, the questionnaire outcomes indicated that the CRC patients in this study reported higher scores on I.ROC compared to the general Dutch population [17], and PH42 outcomes suggested greater resilience, autonomy, and well-being compared to the general Dutch population. Together, these quantitative and qualitative insights illustrate that, although fatigue was prominent according to the CIS, it did not fully capture patients’ overall functional and psychosocial status, which was experienced more positively than the reference group.

It is important to acknowledge that although the psychometric properties of the CIS, PH42, and I.ROC are well established, they have not been validated in a Dutch CRC population. To provide appropriate benchmarks, we compared PH42 and I.ROC scores with data from the general Dutch population (LISS panel [17]), and CIS scores with the Dutch ROM reference group [16]. These comparisons offer relevant cultural and clinical reference points and therefore support the interpretation of our findings, even in the absence of CRC specific psychometric validation.

Patients described the follow-up phase as emotionally demanding due to uncertainty and fear of cancer recurrence. Preferences varied from reassurance through extra tests to comfort in structured schedules. Emotional support and relational continuity are essential in this phase, beyond task-based follow-up [34]. Information breakdowns, particularly between hospital teams and general practitioners, further complicated this phase. Delays in discharge communication mirror systemic gaps and underscore the need for stronger coordination across care boundaries [35]. Addressing them requires clearer role definitions and more standardized information flows to ensure interface continuity beyond the hospital setting.

### Barriers to customization in a modular system

The JBZ CRC pathway has an inherent modular setup, with components such as prehabilitation and follow-up consultations organized as separate service modules. This structure supports efficiency through standardization [36], but customization remains limited. Most decisions are predefined, meaning that the modular design does not yet enable true modular *functioning*.

A key insight from our interviews is that healthcare professionals appear to have limited awareness of the potential of modularity, as they do not think in modular terms. Consequently, the potential of modularity to support flexible, patient-tailored care is not fully realized. Modularity is not only a structural feature but also a cognitive and organizational orientation. Both patients and professionals identified opportunities for greater customization, such as more flexible scheduling, yet realizing these opportunities requires greater professional awareness of modular thinking and clearer role division across modules [21]. Awareness of the full range of treatment and support options offered by different professionals is essential to prevent gaps or overlap in care. In this study, care modules, components, and interfaces were visualized in a modular service architecture (S3 Fig), which enhances our analytical understanding and the potential for personalization. While this visualization was not shared with healthcare professionals or patients, doing so could support awareness of modular options and facilitate shared decision-making (also in other healthcare contexts). Providing patients with a clear overview of available options, aligned with their individual needs and preferences, supports active participation in treatment decisions and emphasizes both medical and personal aspects of care. Modular thinking can be further cultivated through structured reflection on modular options during team meetings, decision support tools that visualize alternative care pathways, and training activities that help professionals in recognizing modules, components, and interfaces in care processes.

### Limitations and strengths

The single centeredness of this case study inherently limits direct generalizability, but it did allow for an in depth exploration of how patient and professional experiences relate to care modularity, albeit within one organizational context. Participants were largely unfamiliar with modularity as a formal concept and described care from their own perspectives, yet their accounts could be interpreted through a modular lens. The mixed methods approach is a notable strength of the study; it provided both breadth and depth by integrating patient experience data with qualitative insights from patients and healthcare professionals. The successful application of the composite Bartels et al. [7] model indicates its potential usefulness for analyzing CRC and other care pathways; however, its generalizability depends on validation in additional cases to explore its applicability across diverse populations and care settings. Importantly, this potential lies not in the direct transferability of empirical findings, but in the model’s function as an analytical framework. The composite Bartels et al. [7] model offered a shared language to map and discuss modular care structures across different organizational contexts, including smaller hospitals or settings without nurse practitioners. While variations in staffing and role distribution may shape how modularity is enacted in practice, the framework itself remains applicable and can support efforts to enhance flexibility and personalization of care across settings.

### Key message

Our study demonstrates that the composite theoretical model developed by Bartels et al. [7], which encompasses the potential of service modularity as a foundation for person-centered care delivered in a shared decision-making context, offers a robust and practical framework. It reveals how limited modular thinking and structural misalignments constrain genuine shared decision-making and customization. Strengthening modular awareness and embedding flexible, patient-tailored approaches throughout the pathway can enhance person-centeredness. This integrative perspective connects modular service design with patient engagement and provides concrete guidance for improving cancer care delivery.

## Supporting information

S1 File Interview guide for patients and healthcare professionals. Semi-structured interview guides used for patient and healthcare professional interviews.
(DOCX)

S2 Table Coding schemes for patient and healthcare professional interviews. Final coding schemes for patient interviews (Table S1) and healthcare professional interviews (Table S2).
(DOCX)

S3 Fig Detailed visualization of the current CRC pathway. Schematic overview of the current CRC pathway as identified in this study.
(PDF)
