# Supplementary material for: Person-centered care, shared decision-making, and service modularity in colorectal cancer treatment: A mixed-method study of patient and professional perspectives
Source: PLoS One. 2026 Mar 6;21(3):e0343331. doi: 10.1371/journal.pone.0343331 (PMC12965563; doi:10.1371/journal.pone.0343331)
Supplement: S1 File — Semi-structured interview guides used for patient and healthcare professional interviews. (DOCX) [file pone.0343331.s001.docx]

S1 File. **Interview guide for patients and healthcare professionals**. Semi-structured interview guides used for patient and healthcare professional interviews.

*Patients*

**Introduction**
Mr or Ms [name], first of all I would like to thank you for your time. I will briefly introduce myself and explain the purpose of the study. My name is [name] and I am a researcher. I am conducting research on the organization of care for people with colorectal cancer at the Jeroen Bosch Hospital (JBZ). The aim of this study is to improve the organization of care, so that the patient is central and the JBZ can provide tailored care. In this interview I will ask you about your experiences and satisfaction, and for example whether you felt that anything was missing in your care. We will use a timeline so that we can discuss this per phase. We will start with some practical questions, then we will talk about how your care process has progressed. Finally, we will discuss how you are doing now. The results of this interview will be treated confidentially and processed anonymously. I would like to ask your permission to record the interview so that I can process the results accurately. Do you have any questions before we continue?

**General questions**

1. Approximately when was the last time you visited the JBZ?
2. When was your most recent treatment?
3. Does someone usually accompany you to your appointments at the JBZ?
   a. Does this help you remember information and possibly make decisions?
4. Are you currently being treated in other departments within the JBZ?

Explanation of the timeline
I would like to create a timeline together with you. As you can see, I have already placed “Suspicion” and “Now” on the timeline. By “Suspicion” I mean the moment when you first thought that something was wrong. Of course, a lot has happened in between. During the interview we will fill in the timeline together. Would you prefer to write yourself, or would you like me to do that?

**Phases**

- Suspicion
- First hospital visit
- Diagnosis
- Treatment plan
- Treatment or treatments
- Aftercare

**Topics to be discussed in each phase**

- Course of events
- Preparation by the JBZ
- Feelings
- Guidance and support
- Information and information transfer
- Ratings

*Tailored care*

- Nurse specialist
- Adaptation to personal wishes and needs
- Sufficient personal attention, such as psychosocial support or peer support groups
- Number of doctors and nurses involved
- Attitude and behavior of doctors and nurses
- Point of contact for questions and complaints
- Shared decision making

*Information provision*

- Information and information transfer
- MijnJBZ (personal patient portal of the JBZ)
- MediMapp (app programmed to guide the patient throught the CRC pathway journey)

*Closing topics*

- Satisfaction
- Most difficult phase or phase with the greatest impact
- Best aspect of the current care process
- Main area for improvement in the current care process

**Standard questions for each phase**

1. How did this phase proceed?
2. Were you well prepared for this phase?
   a. Did the hospital tell you what to expect or how to prepare for your visit?
3. How did you feel during this phase?
   a. What score would you give to how you felt on a scale from 1 to 10 during this phase, emotionally and physically?
4. How did you experience the guidance and support from the hospital during this phase?
5. Did you receive information from the hospital during this phase?
   a. How did you experience the way the information was communicated?
   b. How would you prefer to receive information, for example via a video, app, brochure, or similar?
   c. What score would you give the hospital on a scale from 1 to 10 for this phase?

**Phase specific questions**
**Treatment plan**

1. Did you feel that you were able to make a choice regarding your treatment?
   a. Did you know which options were available?
   b. Did you receive sufficient advice and information to be able to make a choice?
   c. Did you feel supported in making these choices?
   d. Do you feel that you made an informed decision?
   e. Are you still satisfied with your decision?
   f. Did you feel that you were listened to?

**Now**

1. How are you doing now?
2. How do you view the future or the time ahead of you?

**General questions**

*Tailored care*

1. What did the nurse specialist mean to you during the entire care process?
   a. Do you feel that the nurse specialist knows you and your loved ones?
   b. Do you feel that the nurse specialist is aware of your needs and is well acquainted with you as a person and with your loved ones?
2. Did you feel that your personal wishes and needs were taken into account throughout the care process, from scheduling appointments to choosing a treatment?
3. Approximately how many doctors, nurses, and other care providers did you see during your care process?
4. What did you think of the attitude and behavior of the care providers you were in contact with?
   a. Did you feel that the care provider understood you?
   b. Did you understand the care provider?
5. Do you know who to contact at the JBZ with questions or complaints?
6. Do you think the hospital paid sufficient attention to you as a person, for example by offering a conversation with a psychologist or a peer support group?

***General questions on information provision***

1. During your care process you received information on several occasions. How did you experience this information?
   a. How did you experience the way the information was communicated?
   b. How would you prefer to receive information, for example via a video, app, brochure, or similar?
   c. Was the information you received aligned and consistent?
   d. Was the information provided at the right time?
2. Are you familiar with MijnJBZ?
   a. Do you use it?
   b. What do you think of it?
3. Are you familiar with MediMapp?
   a. Do you use it?
   b. What do you think of it?

**Closing**

1. Are you satisfied with the care you received for your colorectal cancer?
   a. Why or why not?
2. Which phase had the greatest impact on you or was most important to you?
3. Which phase was the most difficult for you, and why?
   a. What did you miss?
   b. What would you have preferred?
   c. Where would you have preferred this?
   d. How did you feel?
4. Which of these phases did you find the least difficult, and why?
5. What do you think is the best aspect of the current care process?
6. What do you think is the most important area for improvement in the care process?

We have now reached the end of the questions I wanted to ask you. Do you have any further questions or comments? Thank you very much for your time and participation. Would you like to be informed about the results of the study? Feedback of results: Yes or No. Email address.

*Healthcare professionals*

**Introduction**
[name], first of all I would like to thank you for your time. I will briefly introduce myself and explain the purpose of the study. My name is [name] and I am a researcher. The aim of my research is to improve the health and wellbeing of patients with colorectal cancer by organizing care in a modular way. I focus on the organization of the care process across all stages of rectal and colon carcinoma. A modular care process makes tailored care easier, allowing the patient to be even more central. In this interview I will ask questions about the organization of the care process, its strengths, and potential areas for improvement. We will start with some practical questions and then move on to the care process itself. The results of this interview will be treated confidentially and processed anonymously. I would like to ask your permission to record the interview so that I can process the results accurately. Do you have any questions before we continue?

**Topic list**

- Background of the healthcare professional.
- Role and responsibilities related to colorectal cancer care, including the number and type of patient appointments.
- Collaboration with other departments, such as for comorbidity or geriatrics, collaboration with the Verbeeten Institute (radiotherapy), and collaboration with the nurse specialist.
  Communication with colleagues and how this takes place, for example via email, HiX (electronic health record system), or regular or irregular meetings.
- Communication with patients, including preparation for consultations or treatment, information provision, suitability of information in terms of completeness, amount, timing and format such as brochures or videos, guidance and support, adaptation of attitude, behavior and language to the individual patient, and the use of MijnJBZ and MediMapp.
- Tailored care, including patient freedom of choice, availability of information to support decision making, and completeness of care with regard to positive health and wellbeing.
- Overall organization of care from the patient perspective, including completeness, strengths and areas for improvement.

General questions

1. What is your current role?
   a. Which department or departments do you work in?
   b. Which oncological care pathways or care processes are you involved in?
2. How long have you been working in healthcare?
3. How long have you been working at the JBZ?

**Organization of care**

1. How do you feel care for colorectal cancer is organized?
   a. What do you think works well?
   b. What could be improved?
2. What do you think of the collaboration between the different specialties and departments within the JBZ?
   a. How do transitions between departments take place?
   b. How do transitions between professionals take place?
   c. Do you think the nurse specialist plays an important role in this?
3. What do you think of the collaboration with the Verbeeten Institute?
   a. How does contact with Verbeeten proceed?

**Information**

1. Do you feel that patients are well prepared for the different phases such as diagnostics, treatment and aftercare?
   a. Before each phase, is the patient informed about what to expect or how to prepare for the visit?
2. What type of information does the patient receive during the care process?
   a. What do you think of this information?
   b. How is this information communicated?
   c. Is the information aligned and consistent?
   d. Do you feel the information is provided at the right time?
3. What do you think of MijnJBZ?
   a. Is it being used?
4. What do you think of MediMapp?
   a. Is it being used?

**Guidance and support**

1. What information do you already have about a patient at a first encounter?
   a. How do you obtain this information?
2. To what extent do you adapt your attitude, behaviour and language to each patient?
   a. Do you feel that patients always understand you well?
   b. Do you always understand the patients well?
3. To what extent are you aware of the wishes and needs of the patient and their relatives?
4. What would be different for the patient if you or your role did not exist?
5. What do you think of the guidance and support provided to patients by the hospital?

**Patient autonomy**

1. To what extent do patients have a say in the choice of treatment?
   a. Do patients know which options are available?
   b. Are patients supported in making these choices?
2. To what extent are the personal wishes and needs of the patient taken into account during the care process, from scheduling appointments to choosing a treatment?
3. Do you think there should be more freedom of choice for patients?

**Completeness of care**

1. Is attention paid to the mental health of the patient during the care process?
   a. Are you satisfied with this, and why or why not?
2. Is attention paid to quality of life during the care process?
   a. Are you satisfied with this, and why or why not?
3. Is attention paid to the patient’s daily functioning during the care process?
   a. Are you satisfied with this, and why or why not?
4. Do you think anything is missing in the current care process for people with colorectal cancer?
   a. If so, what?
5. Are there things you should or would like to do for the patient but cannot, for example due to lack of time?
6. If time and or money were not an issue, are there things you would like to do for the patient that are currently not possible?

**Closing**
We have now reached the end of the questions I wanted to ask you. Do you have any questions and or comments? Thank you very much for your time and participation. Would you like to be informed about the results of the study? Feedback of results: Yes or No. Email address.
