# Supplementary material for: Person-centered care, shared decision-making, and service modularity in colorectal cancer treatment: A mixed-method study of patient and professional perspectives
Source: PLoS One. 2026 Mar 6;21(3):e0343331. doi: 10.1371/journal.pone.0343331 (PMC12965563; doi:10.1371/journal.pone.0343331)
Supplement: S2 Table — Final coding schemes for patient interviews (Table S1) and healthcare professional interviews (Table S2). (DOCX) [file pone.0343331.s002.docx]

S2 Table. **Coding schemes for patient and healthcare professional interviews**. Final coding schemes for patient interviews (Table S1) and healthcare professional interviews (Table S2).

*Patients*

**Table S1 Coding scheme patients**

| Psychosocial | |
| --- | --- |
| PS: Uncertainty | The patient indicates experiencing uncertainty. |
| PS: Trust | The patient indicates having trust in the treatment or care trajectory. |
| PS: Coping | The patient shows certain feelings, perceptions, or actions to cope with what is happening. |
| PS: Mental and physical impact | The patient indicates experiencing certain emotions or physical consequences related to the impact of the trajectory or treatment. |
| PS: Resilience | The ability to recover from stress and adversity, mentally and physically. |
| PS: Involvement of relatives | The family or close relatives of the patient are, or are not, actively involved in the care trajectory. |
| Person-centered care | |
| PCC: Attention to functioning | Attention is, or is not, paid to the patient’s daily, social, and societal functioning. |
| PCC: Attention to mental wellbeing | Attention is, or is not, paid to the patient’s mental wellbeing. |
| PCC/SM: Appointment planning | When planning appointments, the preferences of the patient are, or are not, taken into account. |
| PCC: Attitude of healthcare professionals | The patient expresses an opinion about the attitude, approach, and language used by healthcare professionals. |
| PCC: Involvement (personalization) | Involvement of healthcare professionals. |
| PCC: Professional competence of healthcare professionals | Professionally competent healthcare staff are of value to the patient |
| Shared decision-making | |
| SDM: Treatment options explained | The different treatments or options are, or are not, explained with their advantages and disadvantages. In other words, whether the options are truly discussed in depth. |
| SDM: Treatment choice | The patient does, or does not, have freedom in making a treatment choice, or makes the treatment choice together with the healthcare professional. In other words, whether options are presented as real choices. |
| Service Modularity | |
| SM/PCC: Tailored care plan (customization) | The presence, or absence, of various choice options within the care trajectory. In other words, whether the treatment plan is tailored to the patient’s specific situation. |
| SM: Treatment trajectory followed according to an individual plan | The patient indicates whether the treatment trajectory did, or did not, proceed according to the agreed individual plan. |
| SM: Coordination between involved healthcare professionals in secondary care | The patient’s experiences regarding coordination of care within secondary care. |
| SM: Coordination between primary and secondary care | The patient’s experiences regarding coordination between primary and secondary care. |
| Information provision and primary point of contact | |
| Inf: Appreciation, usefulness, satisfaction with the information folder | The patient indicates appreciation, usefulness, or satisfaction regarding the content of the information folder and how it functions. |
| Inf: Appreciation, usefulness, satisfaction with the nurse specialist as point of contact | The patient indicates appreciation, usefulness, or satisfaction regarding the role of the nurse specialist as the primary point of contact for questions or comments. |
| Inf: Appreciation, usefulness, satisfaction with digital information provision | The patient indicates appreciation, usefulness, or satisfaction regarding digital information provision, such as the patient portal, MijnJBZ, or MediMapp. |
| Inf: Appreciation, usefulness, satisfaction with other information provision | The patient indicates appreciation, usefulness, or satisfaction regarding other forms of information provision. |
| Prehabilitation | |
| Prehab: Appreciation, usefulness, satisfaction with prehabilitation | The patient indicates appreciation, usefulness, or satisfaction with the prehabilitation program. |
| Prehab: Physical fitness and activity | The patient indicates to what extent overall physical fitness or activity has been addressed or changed. |
| Prehab: Nutrition | The patient indicates how nutritional status has been addressed or changed. |
| Prehab: Peer support | The patient has, or has not, experienced peer support during participation in the prehabilitation program. |

*Healthcare professionals*

**Table S2 Coding scheme healthcare professionals**

| Algemeen | |
| --- | --- |
| Alg: Quality of care, organizational aspect | The healthcare professional provides suggestions for improving the quality of care with regard to organizational aspects. |
| Alg: Quality of care, clinical content aspect | The healthcare professional provides suggestions for improving the quality of care with regard to clinical content. |
| Information provision | |
| Inf: General communication between healthcare professionals and patient | The general communication between the patient and the healthcare professional is described. |
| Inf: Tailored information provision | Information provision is tailored to the patient. |
| Inf: Content related tasks per role within the care pathway | Substantive tasks per role within the care pathway. |
| Inf: Expectation management | Expectations regarding the care pathway and associated waiting times, diagnostics, and treatments are discussed with the patient. |
| Inf: Appreciation, usefulness, satisfaction with the information folder | The healthcare professional indicates appreciation, usefulness, or satisfaction regarding the content and functioning of the information folder. |
| Inf: Appreciation, usefulness, satisfaction with digital information provision | The healthcare professional indicates appreciation, usefulness, or satisfaction regarding digital information provision, such as the patient portal, MijnJBZ, or MediMapp. |
| Inf: Appreciation, usefulness, satisfaction with other information provision | The healthcare professional indicates appreciation, usefulness, or satisfaction regarding other forms of information provision. |
| Inf: Appreciation, usefulness, satisfaction with the nurse specialist as point of contact | The healthcare professional indicates appreciation, usefulness, or satisfaction regarding the role of the nurse specialist as primary point of contact or case manager. |
| Person-centered care | |
| PCC: Involvement (personalization) | Involvement of healthcare professionals. |
| PCC: Quality of life | The healthcare professional discusses the patient’s quality of life. |
| PCC: Proactive care planning | Proactive care planning is, or is not, used, related to advance care planning. |
| PCC/PS: Attention to functioning | Attention is, or is not, paid to the patient’s daily, social, and societal functioning. |
| PCC/PS: Attention to mental wellbeing | Attention is, or is not, paid to the patient’s mental wellbeing. |
| PCC/SM: Appointment planning (customization) | When planning appointments, the preferences of the patient are, or are not, taken into account. |
| Prehabilitation | |
| Prehab: Physical fitness and activity | The healthcare professional indicates appreciation, usefulness, or satisfaction regarding physical fitness as addressed through prehabilitation. |
| Prehab: Peer support | The healthcare professional indicates appreciation, usefulness, or satisfaction regarding peer support through prehabilitation. |
| Prehab: Nutrition | The healthcare professional indicates appreciation, usefulness, or satisfaction regarding nutrition within prehabilitation. |
| Prehab: Appreciation, usefulness, satisfaction with prehabilitation | The healthcare professional indicates appreciation, usefulness, or satisfaction with the prehabilitation program. |
| Psychosocial | |
| PS: Involvement of relatives | The healthcare professional does, or does not, support the importance of family or relatives being actively involved in the care trajectory. |
| PS: Support services | Referral to internal or external support services does, or does not, take place, actively or otherwise. |
| PS: Trust | The healthcare professional has insight into the patient’s trust in the treatment or care trajectory. |
| Shared decision-making | |
| SDM: Treatment choice | The patient does, or does not, have freedom in making a treatment choice, or makes the treatment choice together with the healthcare professional. In other words, whether options are presented as real choices. |
| SDM: Treatment options explained | The different treatments or options are, or are not, explained with their advantages and disadvantages. In other words, whether the options are thoroughly discussed. |
| Service Modularity | |
| SM: Coordination between involved healthcare professionals in secondary care | The healthcare professional’s experiences regarding coordination of care within secondary care. |
| SM: Coordination between primary and secondary care | The healthcare professional’s experiences regarding coordination between primary and secondary care. |
| SM: Alignment of working methods between healthcare professionals | Working methods within the care pathway are, or are not, aligned between involved healthcare providers. |
| SM: Chemoradiotherapy, treatment phase | The chemoradiotherapy treatment phase within the care pathway is identified in the interview through the coder’s expertise. |
| SM: Chemotherapy, treatment phase | The chemotherapy treatment phase within the care pathway is identified in the interview through the coder’s expertise. |
| SM: Surgery, treatment phase | The surgical treatment phase within the care pathway is identified in the interview through the coder’s expertise. |
| SM: Diagnostic phase | The diagnostic phase within the care pathway is identified in the interview through the coder’s expertise. |
| SM: Follow-up | The follow up phase within the care pathway is identified in the interview through the coder’s expertise. |
| SM: Interfaces | An interface within the care pathway is identified in the interview through the coder’s expertise. |
| SM/PCC: Tailored care delivery | Care delivery is, or is not, adapted to the individual patient. |
