# Supplementary material for: Person-centered care, shared decision-making, and service modularity in colorectal cancer treatment: A mixed-method study of patient and professional perspectives
Source: PLoS One. 2026 Mar 6;21(3):e0343331. doi: 10.1371/journal.pone.0343331 (PMC12965563; doi:10.1371/journal.pone.0343331)

Modular service architecture: Current CCP

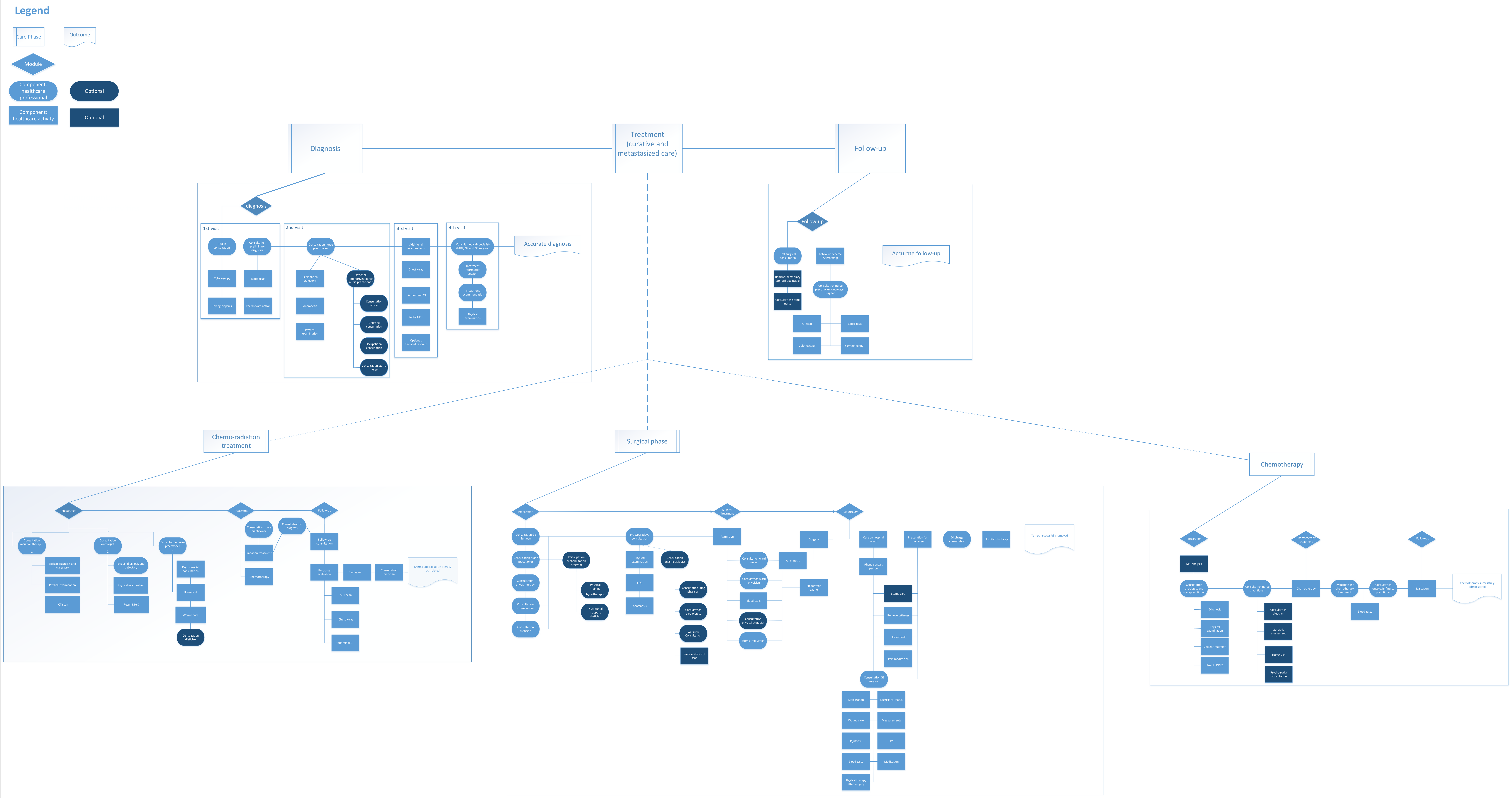

Supplement: S3 Fig — Schematic overview of the current CRC pathway as identified in this study. (PDF) [file pone.0343331.s003.pdf]
